# Downregulated miR‐144‐3p contributes to progression of lung adenocarcinoma through elevating the expression of *EZH2*

**DOI:** 10.1002/cam4.1714

**Published:** 2018-10-02

**Authors:** Chao Liu, Zuozhang Yang, Zhiyong Deng, Youjun Zhou, Quan Gong, Ruilian Zhao, Ting Chen

**Affiliations:** ^1^ Department of Nuclear Medicine Tumor Hospital of Yunnan Province The Third Affiliated Hospital of Kunming Medical College Kunming Yunnan China; ^2^ Departments of Orthopaedics Tumor Hospital of Yunnan Province The Third Affiliated Hospital of Kunming Medical College Kunming Yunnan China; ^3^ Department of Nuclear Medicine The Affiliated Yan'an Hospital of Kunming Medical College Kunming Yunnan China; ^4^ Department of Palliative Medicine Tumor Hospital of Yunnan Province The Third Affiliated Hospital of Kunming Medical College Kunming Yunnan China; ^5^ Departments of Combination of Chinese Traditional and Western Medicine Tumor Hospital of Yunnan Province The Third Affiliated Hospital of Kunming Medical College Kunming Yunnan China

**Keywords:** *EZH2*, lung adenocarcinoma, miR‐144‐3p

## Abstract

**Objectives:**

The intention of our study was to investigate the relationship between miR‐144‐3p and EZH2 as well as the effects of their interaction on cell propagation and invasiveness in lung adenocarcinoma (LUAD).

**Methods:**

The expression levels of miR‐144‐3p and *EZH2* in LUAD tissues and normal tissues were determined by qRT‐PCR. The dual‐luciferase reporter assay was utilized to validate the targeting relationship between miR‐144‐3p and *EZH2*. MTT assay and colony formation assay were performed to evaluate the viability and propagation of LUAD cells, while the effects of miR‐144‐3p and *EZH2* on LUAD cell invasiveness were confirmed by transwell assay. Protein expression levels of *VEGFA*,* MMP2,* and *MMP9* were measured by Western blot. Furthermore, xenograft tumor models were established to verify the effects of miR‐144‐3p on tumor formation and *EZH2*,* VEGFA*,* MMP2* and *MMP9* expressions in vivo.

**Results:**

miR‐144‐3P was downregulated in LUAD tissues, and overexpression of miR‐144‐3p inhibited propagation and invasiveness of LUAD cells. *EZH2* was a target of miR‐144‐3p and was highly expressed in LUAD cells. Knockdown of *EZH2* could suppress the propagation and invasion of LUAD cells. Increased miR‐144‐3p expression exerted an inhibitory effect on LUAD tumor formation in vivo.

**Conclusion:**

Overexpression of miR‐144‐3p impeded the propagation and invasiveness of LUAD cells by targeting *EZH2*.

## 1 INTRODUCTION

Lung adenocarcinoma (LUAD), the most common histologic subtype of lung cancer, accounts for 40% of all diagnosed lung cancer cases.^1^ Despite continuous advances in technology of molecular diagnosis and target therapy, the 5‐year overall survival remains dismal in the clinical management of LUAD.^2^ Currently, the most effective therapy for LUAD is complete surgical resection, while many patients are not diagnosed until metastasis or advanced stages.^3^ Thus, it is imperative to identify suitable molecular markers for LUAD diagnosis and prognosis.

MicroRNA (miRNA) is a small noncoding RNA molecule (containing about 22 nucleotides) which lacks protein‐coding capability and mediates post‐translational regulatory mechanism, regulating a variety of physiological functions and pathological processes.^4^ Many miRNAs have been reported to regulate lung cancer progression. For example, miR‐193 impeded cell invasion and migration in non‐small cell lung cancer.^5^ miR‐454, as a prognostic factor, contributed to lung cancer cell propagation and metastasis.^6^ The role of miR‐144 as a tumor suppressor has been unveiled in several researches regarding human cancers.^7^ Liu et al^8^ verified that miR‐144 was significantly downregulated in gastric cancer tissues. Li et al^9^ demonstrated that miR‐144‐3p arrested cell cycle and stimulated apoptosis in pancreatic cancer cell by targeting proline‐rich protein 11 via the mitogen‐activated protein kinase signaling pathway. In addition, miR‐144 could also inhibit hepatocellular carcinoma cell propagation and metastasis by targeting *ZFX*.^10^ Several studies have reported the involvement of miR‐144 in lung cancer cell activities. For instance, miR‐144 impeded cell proliferation and promoted lung cancer cells apoptosis and autophagy by targeting *TIGAR*.^11^ Gao et al^7^ unraveled that miR‐144 overexpression inhibited non‐small cell lung cancer. However, the specific function of miR‐144 in LUAD remains unclear.

*EZH2* is the functional enzymatic component of polycomb repressive complex‐2 (PRC2) that is involved in health embryonic development through the epigenetic maintenance of genes responsible for regulating development and differentiation.^12^ It has been reported that *EZH2* expression was elevated in cancer tissues in comparison with normal tissues, which contributed to poor prognosis in patients.^13^ Wen et al^14^ discovered that *EZH2* was upregulated in LUAD cells and promoted LUAD cell invasiveness and metastasis. In addition, *EZH2* has been verified to regulate LUAD development through interacting with miR‐26a.^15^ This study evaluated the correlation between miR‐144‐3p and *EZH2* in LUAD to further understanding the mechanism underlying the progression of LUAD.

In this study, qRT‐PCR evaluated the expression level of miR‐144‐3p in LUAD tissues and the impacts of miR‐144‐3p on LUAD cell propagation and invasiveness. Meanwhile, we also verified the target relationship between miR‐144‐3p and *EZH2* and explored the impacts of EZH2 on LUAD cell propagation and invasiveness. At last, we confirmed the effect of miR‐144‐3p on tumor in vivo. Taken together, our study systemically elaborated the molecular network of miR‐144‐3p and *EZH2* in LUAD and provided a deeper understanding of the tumor biology in LUAD.

## 2 MATERIALS AND METHODS

### 2.1 Tissue samples

A total of 31 specimens were collected from lung cancer patients clinically diagnosed during the period from 2014 to 2016 in Tumor Hospital of Yunnan Province, The Third Affiliated Hospital of Kunming Medical College. None of the patients with LUAD received radiotherapy or other treatment before surgery. The resected tissue specimens were stored in a liquid nitrogen tank. Informed written consents were obtained from all subjects. All experiments were performed under the approval of the Ethics Committee of Tumor Hospital of Yunnan Province, the Third Affiliated Hospital of Kunming Medical College.

### 2.2 Cell culture

Human normal pulmonary epithelial cell (BEAS‐2B), human LUAD cells (NCI‐H1975, NCI‐H441, NCI‐H1792 and SPC‐A1), and human embryonic renal cell (293T) were procured from BeNa Biology (Beijing, China). BEAS‐2B cells were cultivated in the LHC‐9 culture (Biofluids Inc., Rockville, MD, USA) with 0.5 ng/mL restructuring epithelial growth factor (EGF), 500 ng/mL hydrocortisone, 0.035 ng/mL bovine pituitary extract, 500 mmol/L of ethanolamine, 500 nmol/L of ethanolamine phosphate, 0.01 mg/mL of adrenaline, and 0.1 ng/mL retinoic acid. NCI‐1975, NCI‐H441, NCI‐H1792, and SPC‐A1 cells were cultured with 10% 0.11 g/L Sodium Pyruvate rpms‐1640 medium (GIBCO BRL, Grand Island, NY, USA), 1.5 g/L NaHCO_3_, and 2.5 g/L glucose.

### 2.3 qRT‐PCR

Samples of total RNA were collected by TRIzol^®^ and rationed by NanoDrop 2000 (Thermo Fisher Inc., Waltham, MA, USA). Reverse transcription was performed using ReverTra Ace qPCR RT Kit (Toyobo, Japan) according to the instructions. The product of reserve transcription was used for quantitative real‐time PCR analysis by means of THUNDERBIRD SYBR^®^ qPCR Mix (Toyobo, Japan). The data were analyzed using 2^−ΔΔCT^. Primer sequences were presented in Table 1.

**Table 1:** Primer sequences for qRT‐PCR

Gene	Sequences
hsa‐miR‐144‐3p	
Forward sequence	5′‐GCGCGCTACAGTATAGATGATG‐3′
Reverse sequence	5′‐GCTGTCAACGATACGCTACG‐3′
EZH2	
Forward sequence	5′‐GTGGAGAGATTATTCTCAAGATG‐3′
Reverse sequence	5′‐CCGACATACTTCAGGGCATCAGCC‐3′
GAPDH	
Forward sequence	5′‐GTCAACGGATTTGGTCTGTATT‐3′
Reverse sequence	5′‐CGCUUCACGAAUUUGCGUGUCAU‐3′
U6	
Forward sequence	5′‐GGAGCGAGATCCCTCCAAAAT‐3′
Reverse sequence	5′‐GGCTGTTGTCATACTTCTCATGG‐3′

### 2.4 Cell transfection

miR‐144‐3p mimics, si‐*EZH2*, negative sequence, *EZH2*‐pcDNA3.1, and pcDNA3 plasmid vector were purchased from GenePharma (Shanghai, China). Cells without any treatment were used as blank group; cells transfected with negative sequence served as NC group; cells transfected with miR‐144‐3p mimics served as miR‐144‐3p group; cells transfected with si‐*EZH2* served as si‐*EZH2* group; cells cotransfected miR‐144‐3P mimics and *EZH2*‐pcDNA3.1 served as mimics+*EZH2* group. During 24 hours before transfection, NCI‐H1975 and APC‐A1 cells at logarithmic growth stage were resuspended in completed medium overnight. The original medium was subsequently replaced with fresh serum‐free medium. Transfection was performed following the guideline of Liposome 2000 (Life Technologies, Carlsbad, CA, USA). Transfection sequence was listed in Table 2.

**Table 2:** Transfection sequence

Name	Sequences
miR‐144‐3p mimics	5′‐UACAGUAUAGAUGAUGUACU‐3′
miR‐144‐3p inhibitor	5′‐AUGUCAUAUCUACUACAUGA‐3′
Mimics control	5′‐UCGCAUGCCACAGCCAUGGC‐3′
si‐EZH2	5′‐CCTGGTCTGGCTTTATGCTAAGTTT‐3′
siRNA control	5′‐CCTGTCTTTCGGTATAATCGGGTTT‐3′

### 2.5 Colony formation assay

Each group cells in logarithmic growth phase were collected and dispersed into single cell suspension and then were added to RPMI1640 culture medium for 1‐2 weeks until duplicated cells were visible. After discarding the supernatant liquid, 4% polyformaldehyde was employed to fix cells and GIMSA (Sigma‐Aldrich, st. Louis, MO, USA) was used for staining for 10‐30 minutes. The quantity of colonies was recorded under the microscope. The experiment was repeated three times.

### 2.6 MTT assay

Cells were incubated with 10 μL MTT solution for 4 hours. After removal of liquid, DMSO was added to per well to dissolve the crystal. The absorbance of each well was measured at the wavelength of 490 nm using a microplate reader.

### 2.7 Transwell assay

Cells were cultured with 50 μL madrigal diluted by serum‐free medium. Cell suspension was obtained after transfection. 200 μL cell suspension (5 × 10^3^ cells/well) was placed in the upper chamber and 500 μL RPMI164 medium containing 10% FBS was added into the lower chamber. Having been washed with PBS twice, the cells were fixed using 4% paraformaldehyde for 20 minutes and stained with 0.1% crystal violet for 30 minutes, followed by being washed with PBS twice. The cells were photographed under a microscope.

### 2.8 Dual‐luciferase reporter assay

*EZH2*‐3′UTR was mutated using multisite‐directed mutagenesis. 3′UTRs (wild and mutated type) were inserted into pmirGLO vector (Promega, Madison, WI, USA). miR‐144‐3p mimics or mocks and *EZH2*‐wt or *EZH2*‐mut 3′UTRs were cotransfected into cells. The activity of luciferase was detected after 48 hours of cultivation.

### 2.9 Western blot

Protein concentration was determined using Pierce BCA Protein Assay Kit (Pierce, Rockford, IL, USA). Having been separated by 10% SDS‐polyacrylamide gel electrophoresis (SDS‐PAGE), proteins were transferred onto the polyvinylidene difluoride membranes (PVDF, Millipore, Burlington, Massachusetts, USA) for 120 minutes. Then, cells were blocked with TBST including 5% nonfat skimmed milk and then incubated with primary antibodies (anti‐*EZH2*, ab186006, 1:1000; anti‐VEGFA, ab46154, 1 μg/mL; anti‐MMP2, ab97779, 1:2000; anti‐MMP9, ab119906, 1:1000; anti‐GAPDH, ab9485, 1:2500; Abcam, Cambridge, MA, USA). Subsequently, secondary antibodies were supplemented to culture the cells (Goat anti‐rabbit IgG H&L, ab6721, 1:10 000; Goat anti‐mouse IgG H&L, ab6789, 1:2000; Abcam). The signal detection was conducted by means of ECL system (Life Technology). GAPDH was regarded as an internal reference.

### 2.10 In vivo xenograft study

Male nude mice, 5‐7 weeks old, were purchased from Shanghai Experimental Animal Center (Shanghai, China). All animal studies were performed in accordance with the guidelines of the Animal Experiments Ethics Committee of Tumor Hospital of Yunnan Province, The Third Affiliated Hospital of Kunming Medical College. Mice were randomized into two groups, 6 mice per group. The mice, served as NC group, were injected subcutaneously with NCI‐H1975 cells transfected with negative sequence, and served as Mimics group, were injected subcutaneously with NCI‐H1975 cells transfected with miR‐144‐3p mimics. Tumor formation was monitored by measuring the tumor volume every 7 days with caliper measurements. After 42 days, all of the mice were sacrificed and tumors were weighted.

### 2.11 Statistical analysis

The measured parameters were presented as means ± SD. GraphPad Prism 7.0 software (La Jolla, CA, USA) and SPSS 24.0 (IBM, Armonk, NY, USA) were used for statistical analysis. Statistical tests for data analysis were two‐tailed *t* test, one‐way ANOVA, and chi‐square tests. Kaplan‐Meier survival analysis and logarithmic rank test were used to compare the postoperative survival of patients. *P *<* *0.05 was considered to be statistically significant.

## 3 RESULTS

### 3.1 miR‐144‐3P was downregulated in LUAD tissues

**Figure 1:**
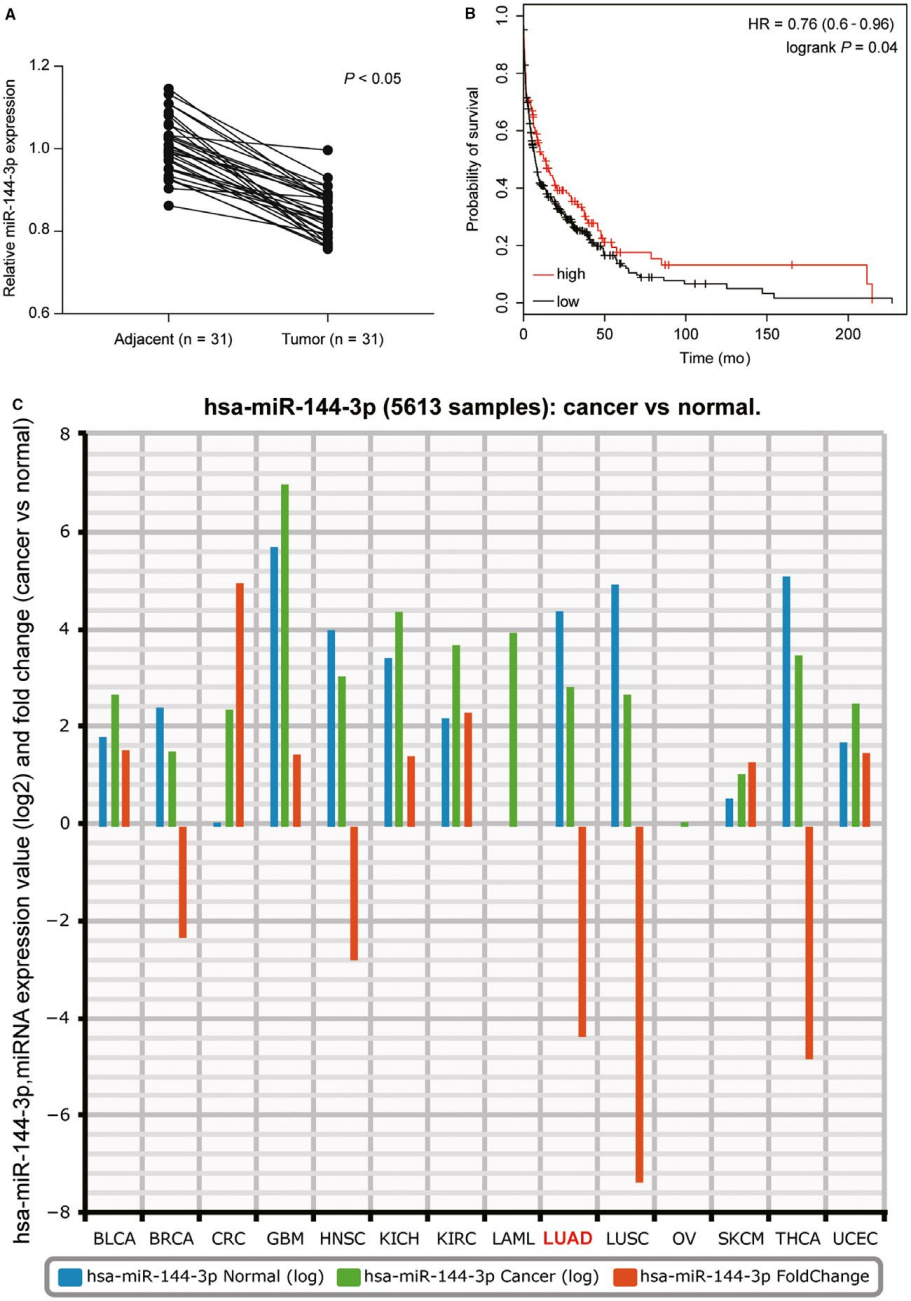
miR‐144‐3p was downregulated in LUAD. A, The expression of miR‐144‐3p in 31 pairs of LUAD tissues was lower than that in adjacent tissues determined by qRT‐PCR. B, The Kaplan‐Meier analysis with TCGA database showed that LUAD patients with low miR‐144‐3p expression had a poor overall survival compared to patients with high miR‐144‐3p expression. C, The result showed miR‐144‐3p expression level in TCGA database was lower in LUAD compared with that in normal through StarBase v2.0

The results manifested that compared with adjacent tissues, miR‐144‐3p was lowly expressed in tumor tissues (Figure 1A, *P* < 0.05). Then, we probed into the correlation between the expression of miR‐144‐3p and the clinical characteristics of patients with LUAD. As shown in Table 3, miR‐144‐3p expression was significantly correlated with TNM staging and lymph node metastasis (*P *<* *0.05) and it had nothing to do with gender or age (*P *>* *0.05). In addition, Kaplan‐Meier analysis with TCGA database indicated that the lower the expression of miR‐144‐3p, the poorer the patients’ survival (Figure 1B). Meanwhile, the results in Figure 1C revealed that the expression level of miR‐144‐3p in TCGA database was lower in LUAD than that in normal.

**Table 3:** Correlation between miR‐144‐3p expression and clinicopathological features in LUAD patients

Parameters	Group	Total	miR‐144‐3p		*P* value
			Low	High	
Gender	Male	21	13	8	0.097
	Female	10	3	7	
Age	<60	19	9	10	0.756
	≥60	12	5	7	
Tumor size	<3 cm	8	6	2	0.260
	≥3 cm	23	12	11	
Differentiation	Well	9	5	4	0.457
	Moderate‐poor	22	9	13	
TNM stage	I+II	15	10	5	0.020^a^
	III+IV	16	4	12	
Lymph node metastasis	Absence	22	8	14	0.036^a^
	Presence	9	7	2	

LUAD, lung adenocarcinoma.

a *P *<* *0.05.

### 3.2 Overexpression of miR‐144‐3p inhibited propagation and invasiveness of LUAD cells

**Figure 2:**
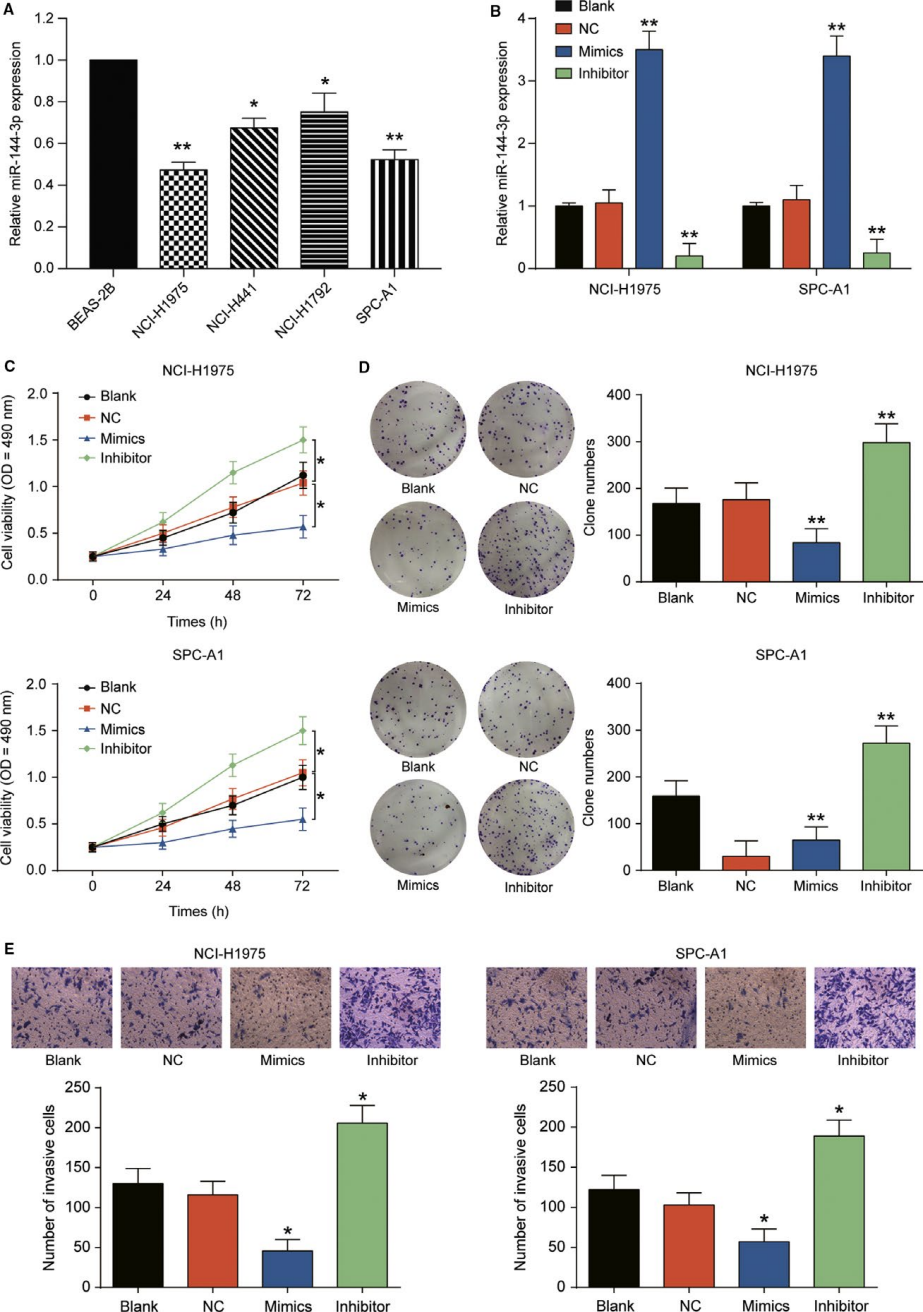
miR‐144‐3p overexpression suppressed LUAD cell proliferation and invasion in vitro. A, The expression of miR‐144‐3p in four LUAD cell lines (NCI‐H1975, NCI‐H441, NCI‐H1792, and SPC‐A1) was lower than that in normal cell line BEAS‐2B. B, miR‐144‐3p mRNA expression in mimics group was much higher than that in NC group. At the same time, the expression level in inhibitor group was much lower than in that in NC group. C, Cell viability in mimics group was lower than that in NC group detected by MTT assay and the cell viability in inhibitor group was higher than that in NC group. D, It was verified that clone number was decreased by miR‐144‐3p mimics and increased by miR‐144‐3p inhibitor through clone formation assay. E, It was found that miR‐144‐3p mimics inhibited cell invasion but miR‐144‐3p inhibitor enhanced cell invasion through transwell assay. *P <* *0.05, **P <* *0.01, compared with NC group

Four different LUAD cell lines were selected to determine the expression of miR‐144‐3p. Compared with normal pulmonary cell lines BEAS‐2B, miR‐144‐3p expression was observably decreased in LUAD cell lines (Figure 2A, *P* *<* 0.01); thus, we chose NCI‐HI975 and SPC‐A1 cell lines for the following analyses. qRT‐PCR indicated that the expression of miR‐144‐3p remarkably increased in mimics group while decreased in inhibitor group in comparison with NC group (Figure 2B, *P* *<* 0.01). MTT assay and colony information assay showed that overexpression of miR‐144‐3p significantly inhibited the propagation of NCI‐H1975 and SPC‐A1 cells, whereas decreased miR‐144‐3p expression significantly promoted the propagation of LUAD cells (Figure 2C,D, *P* < 0.01). Transwell assay indicated that miR‐144‐3p mimics impeded cell invasiveness, while miR‐144‐3p inhibitor enhanced cell invasiveness (Figure 2E, *P* *<* 0.01). The results above suggested that overexpression of miR‐144‐3p inhibited the propagation and invasiveness of LUAD cells.

### 3.3 *EZH2* was the target of miR‐144‐3p and highly expressed in LUAD cells

**Figure 3:**
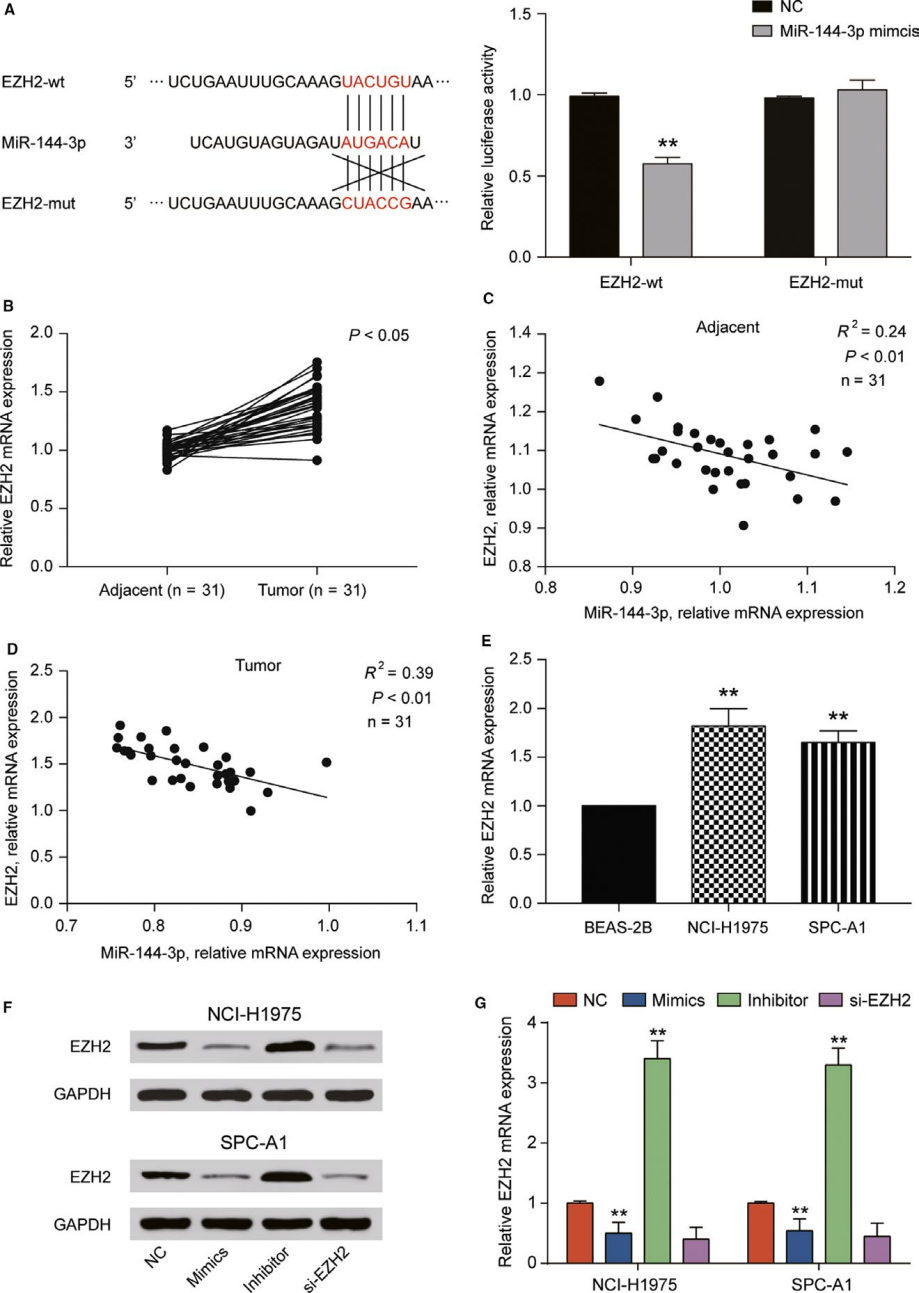
*EZH2* expression was directly regulated by miR‐144‐3p. A, TargetScan predicted that the binding site of miR‐144‐3p existed at the 3′‐UTR region of *EZH2*, and dual‐luciferase reporter assay showed that luciferase activity in *EZH2*‐wt transfected with miR‐144‐3p was lower than that in NC group. **P <* *0.01, compared with NC group. B, The expression of *EZH2* in tumor tissues was higher than that in adjacent tissues determined by qRT‐PCR. C, D, *EZH2* mRNA expression was negatively correlated with miR‐144‐3p mRNA expression in both adjacent tissues and tumor tissues. E, qRT‐PCR analysis indicated that *EZH2* mRNA expression in NCI‐H1975 and SPC‐A1 cell lines was much higher than that in BEAS‐2B cell line. F, G, Expression of *EZH2* in NCI‐H1975 and SPC‐A1 cells transfected with miR‐144‐3p mimics or si‐*EZH2* was lower than that in NC group and the expression after transfected with miR‐144‐3p inhibitor was higher than that in NC group detected by Western blot and qRT‐PCR. **P <* *0.01, compared with BEAS‐2B, compared with NC group

The potential target of miR‐144‐3p was predicted by TargetScan (http://www.targetscan.org). The targeted relationship between *EZH2* and miR‐144‐3p was validated by dual‐luciferase reporter assay (Figure 3A, *P* *<* 0.01). qRT‐PCR assay indicated that *EZH2* expression was elevated in LUAD tissues compared with normal ones (Figure 3B, *P* *<* 0.05). And there was a negative correlation between miR‐144‐3p expression and *EZH2* expression in both adjacent tissues and tumor tissues (Figure 3C,D). We discovered that *EZH2* expression was elevated in NCI‐H1975 and SPC‐A1 cell lines more significantly than in BEAS‐2B cell line (Figure 3E, *P* *<* 0.05). Western blot and qRT‐PCR revealed that si‐*EZH2* and miR‐144‐3p mimics significantly suppressed *EZH2* expression in NCI‐H19575 and SPC‐A1 cell lines, while miR‐144‐3p inhibitor enhanced *EZH2* expression (Figure 3F,G, *P* *<* 0.01). Overall, *EZH2* was a target of miR‐144‐3p and was highly expressed in LUAD cells.

### 3.4 Knockdown of *EZH2* inhibited the propagation and invasiveness of LUAD cells

**Figure 4:**
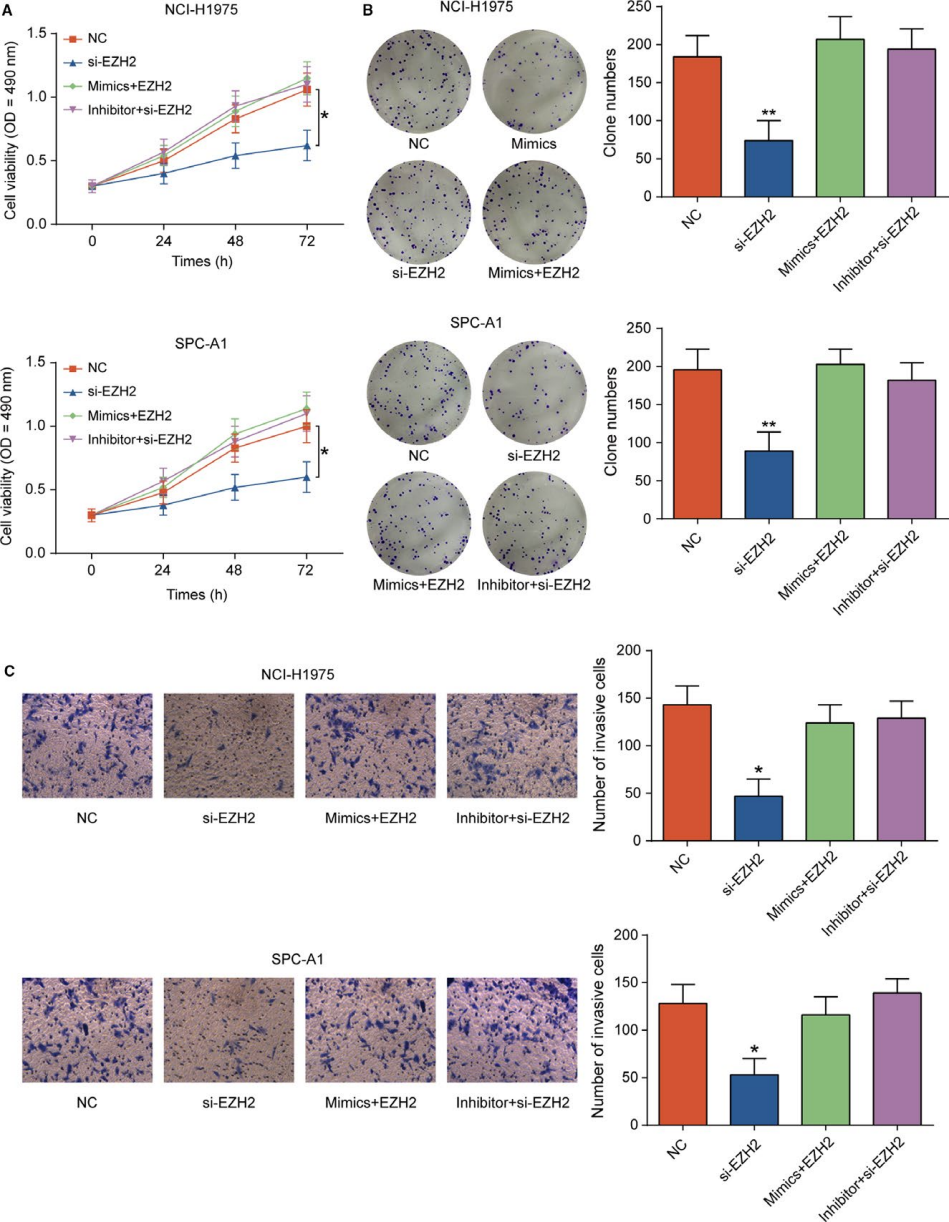
*EZH2* inhibition suppressed LUAD cell proliferation and invasion. A, Cell viability in si‐*EZH2* group was lower than that in NC group detected by MTT assay. And the phenomenon of reduced cell viability caused by *EZH2* inhibition was restored via miR‐144‐3p inhibitor. B, Colony numbers in si‐*EZH2* group were less than that in NC group detected by colony formation assay. miR‐144‐3p inhibitor could restored the colony numbers. C, Effect of si‐*EZH2* on cell invasion of NCI‐H1975 and SPC‐A1 cells was detected by transwell assay, while this phenomenon was restored by cotransfecting miR‐144‐3p inhibitor and *EZH2* inhibition. **P <* *0.01, compared with NC group. Scale bar: 50 μm

**Figure 5:**
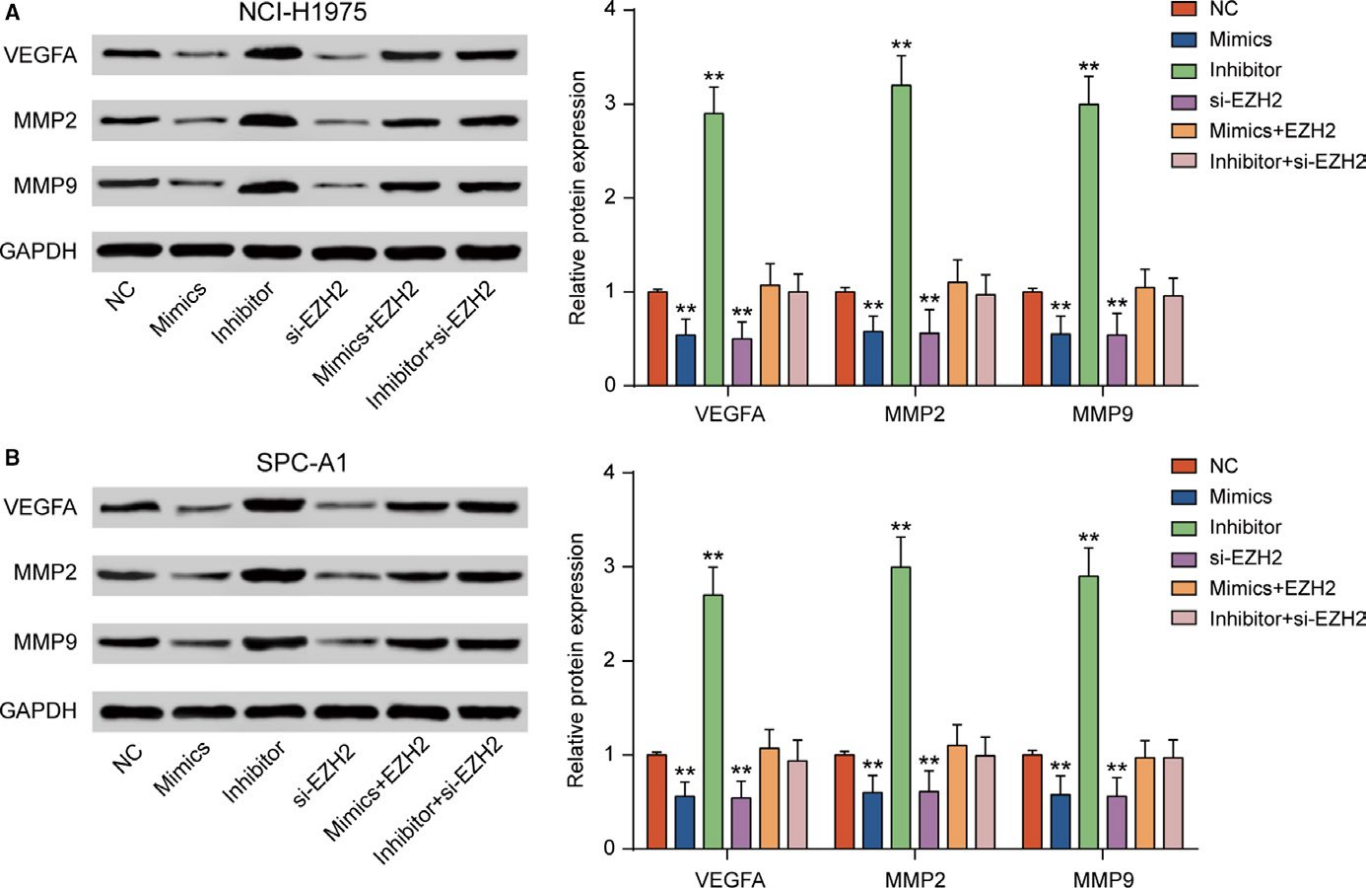
*EZH2* promoted the expression of *VEGFA*,* MMP2,* and *MMP9* in the LUAD cell lines. A, B, *VEGFA*,* MMP2,* and *MMP9* were downregulated in NCI‐H1975 and SPC‐A1 cells transfected with miR‐144‐3p mimics and si‐*EZH2*, but they were upregulated transfected with miR‐144‐3p inhibitor. **P <* *0.01, compared with NC group

MTT assay and colony information assay showed that knockdown of *EZH2* significantly inhibited cell viability and propagation of NCI‐H1975 and SPC‐A1 cells compared with NC group; however, miR‐144‐3p inhibitor could restore cell viability and propagation (Figure 4A,B, *P* < 0.01). Transwell assay exhibited that si‐*EZH2* impaired cell invasiveness (Figure 4C, *P* < 0.05). The expressions of vascular endothelial growth factor (*VEGF*), matrix metalloproteinase (*MMP*)‐2, and *MMP‐9*, which served as tumor promoter, were detected by Western blot. The protein expression of *VEGFA*,* MMP2,* and *MMP9* was restrained in si‐*EZH2* group while restored by miR‐144‐3p inhibitor (Figure 5A,B, *P* *<* 0.05). Taken together, knockdown of *EZH2* suppressed the cell propagation and invasiveness in LUAD.

### 3.5 miR‐144‐3p suppressed tumor growth in vivo

**Figure 6:**
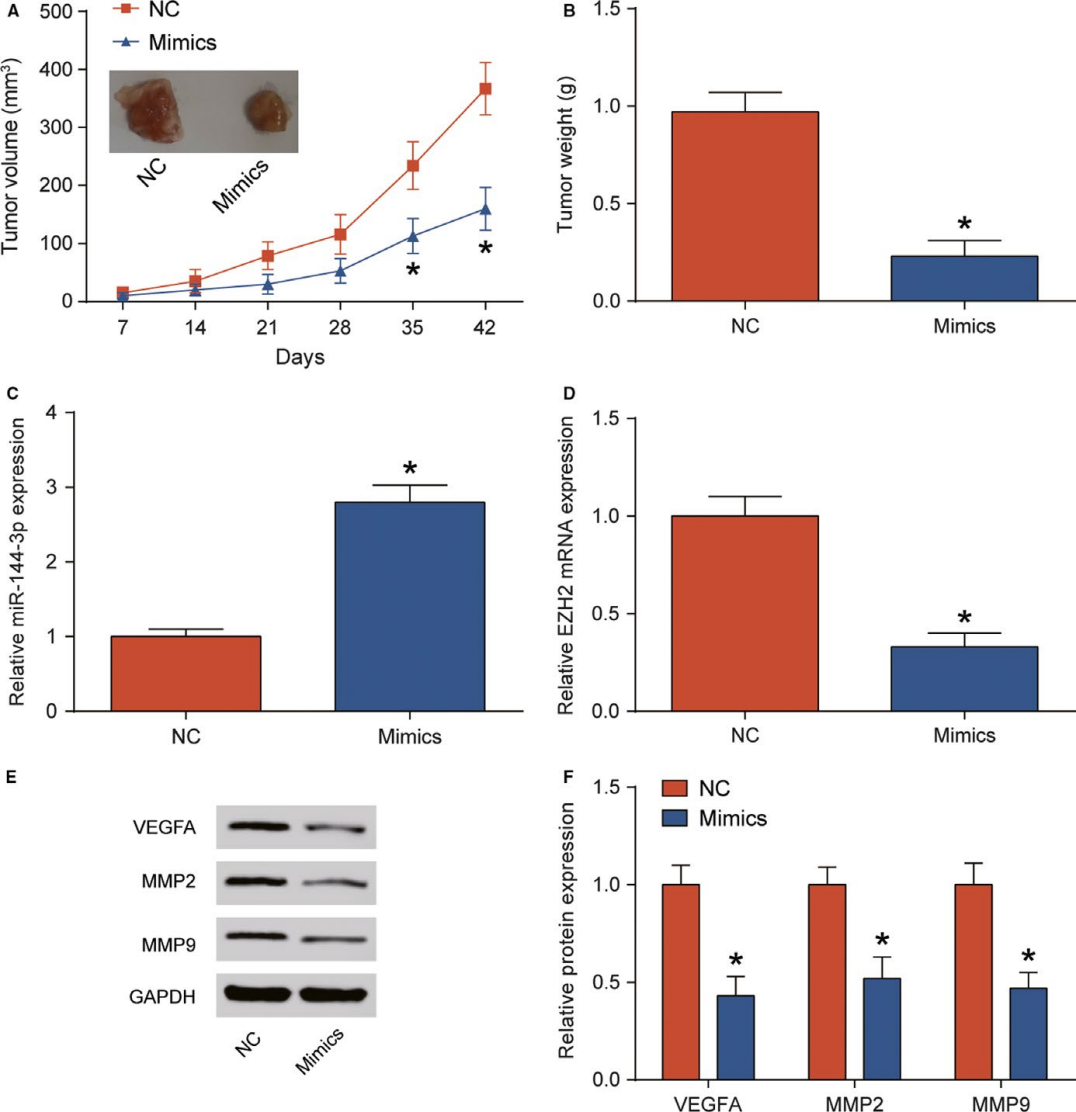
miR‐144‐3p inhibited NCI‐H1975 cells xenograft tumor growth in nude mice. A, miR‐144‐3p suppressed the growth of xenograft tumor volume in nude mice. B, miR‐144‐3p significantly decreased tumor weight in nude mice. Tumors were weighted on Day 42. C, D, qRT‐PCR analysis was used to detected miR‐144‐3p and EZH2 expression level in NC group and miR‐144‐3p mimics group. E, F, The results of Western blot and qRT‐PCR were verified that miR‐144‐3p downregulated *VEGFA*,* MMP2,* and *MMP9* in mimics group. *P <* *0.05, compared with NC group

**Figure 7:**
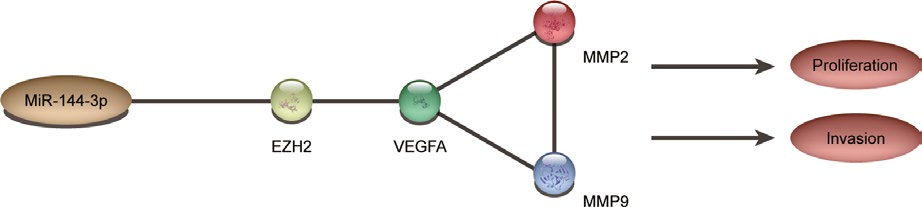
The carton of the mechanism underlying the miR‐144‐3p‐*EZH2*‐*VEGFA*‐*MMP2*/*MMP9* axis in LUAD

To validate whether miR‐144‐3p inhibits LUAD xenograft tumor growth in vivo, NCI‐H1975 cells with stable overexpression miR‐144‐3p or control were injected subcutaneously into nude mice. After 42 days, the tumor volume and weight were significantly decreased in miR‐144‐3p mimics group compared with those in NC group (Figure 6A,B, *P* *<* 0.05). The qTR‐PCR results showed that miR‐144‐3p mimics increased the expression of miR‐144‐3p in nude mice while downregulated *EZH2* expression (Figure 6C,D, *P* *<* 0.05). The effects of miR‐144‐3p on *VEGFA*,* MMP2,* and *MMP9* were confirmed by Western blot (Figure 6E,F, *P* *<* 0.05). All of these results demonstrated that miR‐144‐3p exerted an inhibitory influence on LUAD growth in vivo. The mechanism underlying the miR‐144‐3p, *EZH2*,* VEGFA,* and *MMP2*/*MMP9* axis in LUAD was presented in Figure 7.

## 4 DISCUSSION

Lung adenocarcinoma accounts for a majority of cancer‐related deaths annually.^16^ In current study, we investigated miR‐144‐3p expression and its effect on LUAD cell propagation and invasiveness. Furthermore, we also verified that *EZH2* was a direct target of miR‐144‐3p. MTT assay, colony formation assay, and transwell assay all revealed the effects of the interaction between *EZH2* and miR‐144‐3p on LUAD cellular function. Moreover, the inhibitory influence of miR‐144‐3p on LUAD growth was further confirmed in vivo. Finally, we demonstrated that miR‐144‐3p inhibited LUAD cell propagation and invasiveness via downregulation of *EZH2* expression. Our study firstly clarified the prognostic value of the coexpression of miR‐144‐3p and *EZH2* in LUAD.

In recent years, miRNAs have attracted more attention of researchers due to their crucial role in cell propagation, metastasis and differentiation.^17^ Dysregulation of miR‐144‐3p has been found to be involved in the progression of various human cancers. For example, miR‐144‐3p promoted cell propagation and metastasis in clear cell renal cell carcinoma.^18^ In addition, miR‐144‐3p also serves as a tumor suppressor in glioblastoma by impairing cell propagation, invasiveness, and migration.^19^ It has been reported in previous studies that miR‐144‐3p has complicated regulation mechanism in different types of tumors. In lung cancer, miR‐144 was verified to be significantly downregulated.^20^ Chen et al^21^ corroborated that miR‐144 inhibited propagation and induced apoptosis and autophagy in lung cancer cells by targeting TIGAR. To examine the role of miR‐144‐3p in LUAD cells, we firstly detect the expression level of miR‐144‐3p in LUAD. The results revealed that miR‐144‐3p was downregulated in LUAD tissues. Meanwhile, Kaplan‐Meier analysis manifested that the survival rate of patients with LUAD was positively related with miR‐144‐3p expression. MTT assay, colony formation assay, and transwell assay demonstrated that miR‐144‐3p exerted inhibitory effect on cell viability, propagation, and invasiveness. Furthermore, xenograft study also substantiated that stable overexpression miR‐144‐3p induced by miR‐144‐3p mimics could decrease the expression of *EZH2*, suppressing tumor growth. Our findings provide a novel therapeutic target for patients with LUAD.

*EZH2*, a methyltransferase, has been identified as an oncogene in multiple diseases.^22^ Inhibition of *EZH2* through direct and indirect mechanisms, such as epigenetic activation of oncogenic signaling cascades and silencing of tumor suppressor genes, has been reported to be an important approach for cancer treatment.^23^ For instance, miR‐101 inhibited the metastasis of osteosarcoma cells by downregulation of *EZH2* expression.^24^ Lin et al^25^ demonstrated that miR‐144 suppressed tumorigenesis of astrocytoma by targeting *EZH2*. In our study, *EZH2* was upregulated in LUAD cells and targeted by miR‐144‐3p. To investigate the potential role of *EZH2* in LUAD, we knocked down *EZH2* and discovered that silencing of *EZH2* impeded the propagation and invasiveness of LUAD cells. Analogously, a previous study also indicated that miR‐26a could decrease the propagation and increase the apoptosis rate of LUAD cells by downregulating *EZH2*.^15^ However, few study focuses on the interaction between *EZH2* and miR‐144‐3p in LUAD. Our study provided a new insight for therapy of LUAD. However, some deficiencies in our study are worthy to be noticed. We only investigated the impacts of miR‐144‐3p/*EZH2* on the propagation and invasiveness of LUAD cells, while other cell activities should also be considered, such as apoptosis and autophagy.

In summary, our experiments verified that miR‐144‐3p was downregulated in LUAD tissues, which was functionally associated with the propagation and invasiveness of LUAD cells. These results suggested that miR‐144‐3p may be one of the candidates for new molecular diagnosis and prognosis in LUAD. Besides, *EZH2*, a target of miR‐144‐3p, serves as an oncogene in LUAD. Our study offered a deeper insight into the mechanisms underlying the progression of LUAD and identified miR‐144‐3p as a potential anticancer agent for therapy of LUAD.

## CONFLICT OF INTEREST

None declared.
